# Segmental abnormalities of superior longitudinal fasciculus microstructure in patients with schizophrenia, bipolar disorder, and attention-deficit/hyperactivity disorder: An automated fiber quantification tractography study

**DOI:** 10.3389/fpsyt.2022.999384

**Published:** 2022-12-06

**Authors:** Feiyu Xu, Chengliang Jin, Tiantian Zuo, Ruzhan Wang, Ying Yang, Kangcheng Wang

**Affiliations:** ^1^School of Mental Health, Jining Medical University, Jining, China; ^2^Shandong Mental Health Center, Shandong University, Jinan, China; ^3^Cheeloo College of Medicine, Shandong University, Jinan, China; ^4^School of Psychology, Shandong Normal University, Jinan, China

**Keywords:** schizophrenia, bipolar disorder, ADHD, superior longitudinal fasciculus, automated fiber quantification

## Abstract

**Introduction:**

Superior longitudinal fasciculus (SLF) is a white matter (WM) tract that connects the frontal, parietal and temporal lobes. SLF integrity has been widely assessed in neuroimaging studies of psychiatric disorders, such as schizophrenia (SZ), bipolar disorder (BD), and attention-deficit/hyperactivity disorder (ADHD). However, prior studies have revealed inconsistent findings and comparisons across disorders have not been fully examined.

**Methods:**

Here, we obtained data for 113 patients (38 patients with SZ, 40 with BD, 35 with ADHD) and 94 healthy controls from the UCLA Consortium for Neuropsychiatric Phenomic LA5c dataset. We assessed the integrity of 20 major WM tracts with a novel segmentation method by automating fiber tract quantification (AFQ). The AFQ divides each tract into 100 equal parts along the direction of travel, with fractional anisotropy (FA) of each part taken as a characteristic. Differences in FA among the four groups were examined.

**Results:**

Compared to healthy controls, patients with SZ showed significantly lower FA in the second half (51–100 parts) of the SLF. No differences were found between BD and healthy controls, nor between ADHD and healthy controls. Results also demonstrated that patients with SZ showed FA reduction in the second half of the SLF relative to patients with BP. Moreover, greater FA in patients in SLF was positively correlated with the manic-hostility score of the Brief Psychiatry Rating scale.

**Discussion:**

These findings indicated that differences in focal changes in SLF might be a key neurobiological abnormality contributing to characterization of these psychiatric disorders.

## Introduction

Psychiatric disorders such as schizophrenia (SZ), bipolar disorder (BD), and attention-deficit/hyperactivity disorder (ADHD) are substantial causes of the global growing disability and morality. Among these mental disorders, similar neurocognitive deficits (1) and underlying genetic basis (2, 3) have been found. For instance, differences in brain networks associated with memory and cognitive control have been reported in these disorders, particularly those in prefrontal, default mode, and limbic networks (4, 5). Notably, symptom profiles differ across these disorders, with SZ high in psychotic features, BD increased in emotional dysfunction and ADHD high in externalizing behaviors. Exploring the neuropathological characteristics across psychiatric disorders is critical for understanding their pathophysiology. Brain neuroimaging studies have identified imbalanced connectivity within and between the attention, frontoparietal, default, and reword networks among psychiatric disorders (6). Investigating structural connectivity abnormalities could help understand dysfunction of these brain networks. Structural connectivity refers to brain white matter (WM), which consists of myelinated fiber bundles and structurally connects regions that are not physically proximal. In human brain, major long WM tracts, such as superior longitudinal fasciculus (SLF), are largely responsible for communications between various areas of the cerebral cortex. Disturbances in these tracts or neural networks are considered important etiopathogenetic factors of psychiatric disorders. Previous studies have found altered WM tracts among the three psychiatric disorders, such as the prefrontal lobe, temporal lobe, and anterior corona radiata fiber tract (7–12). However, to what extent these three psychiatric disorders have shared and distinct WM abnormalities remains elusive. There is a need to explore the microstructure of WM integrity across psychiatric disorders.

**FIGURE 1:**
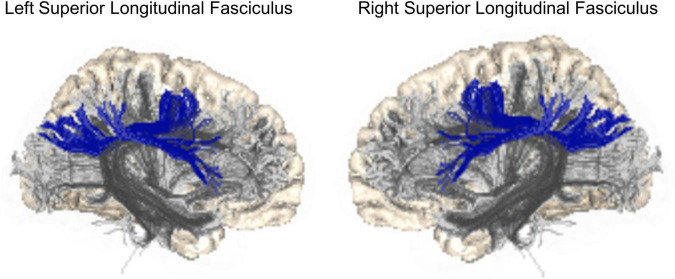
Location of the left and right superior longitudinal fasciculus showing in the brain.

Structural connectivity disturbances are considered an important etiopathogenetic factors of major psychoses (13), including SZ and BD. They were identified within large-scale disconnected brain networks (14). Among WM tracts, the SLF has been widely reported to show reduced fractional anisotropy (FA) in many studies (15–18). SLF is a large WM bundle that connects and allows communication among the frontal, parietal, and temporal lobes (Figure 1). The main function of the SLF consists of attention, executive control, visual and spatial cognition, control of motor processes, and language functions (19). Decreased FA has also been reported in patients at high risk of psychosis (20–25). Prior WM *in vivo* magnetic resonance imaging (MRI) studies of SZ and BD have demonstrated decreased FA in SLF in both disorders (16) with greater heterogeneity in findings of BD (26–28).

Attention-deficit/hyperactivity disorder has also been found related to defects in brain networks involving in cognitive, affective, and motor behaviors (29). Similar to SZ and BD, cognitive deficits of ADHD consist of attention, memory, response control, emotional instability, and impulsive behaviors. Abnormality in the SLF, which showed associations with these symptoms, has been reported in patients with ADHD (30–35). For example, in a longitudinal follow-up study, Cortese et al. (34) suggested that decreased FA may be a lasting characteristic change in patients with ADHD. The decline of FA in SLF continued into adulthood and did not recover as symptoms got better. While Silk et al. (36) found increased FA in the SLF in ADHD patients, some other studies found no significant difference compared to healthy controls (37–41). Further studies are needed to investigate abnormalities in the SLF in ADHD patients.

Previous inconsistent findings in psychiatric disorders might be partly due to methodological differences across studies. The region of interest (ROI) based approach and the tract-based spatial statistics were frequently utilized to analyze Diffusion-weighted images (DWI). The two methods analyze WM microstructures across the whole brain. However, they were of limited utility in investigating the integrity of specific WM tracts. Tractography might be a more suitable method to systematically identify and quantify WM tracts in the human brain. In recent years, Automated Fiber Quantification (AFQ) shows up as an advance in tractography and has been successfully applied into psychiatric disorders. This allows researchers to quantify diffusion measures at sample positions from the start to the end of each reconstructed tract. Some studies have applied AFQ to reveal microstructural changes in WM tracts in SZ, BD, and patients with multiple sclerosis, obsessive-compulsive disorder, and Alzheimer’s disease (42–46). Importantly, all these above studies found that microstructural abnormalities in WM tracts were focal, not entire.

Considering inconsistent findings and fewer comparisons across psychiatric disorders, we used the novel AFQ method to automatically reconstruct WM fibers in patients with SZ, BD, and ADHD. We aimed to identify focal changes of the SLF in these three disorders by comparing healthy controls to each patient group. The shared and distinct alterations of FA in SLF were then explored in patient groups. Correlation analyses were also performed to test whether any focal abnormalities in the patient groups were associated with clinical symptoms. We hypothesized that patients with SZ, BD, and ADHD would show abnormal FA in SLF relative to healthy adults and that differences in FA would be associated with severity of clinical symptoms.

## Materials and methods

### Participants

Participants of this study were from the UCLA Consortium for Neuropsychiatric Phenomics (CNP). The CNP dataset is available *via* the OpenfMRI project^1^ and is comprised of healthy controls and patients with SZ, BD, and ADHD. The detailed information on demographic, clinical, and neuroimaging data of participants has been described in the public data descriptor (1). Both T1-weighted imaging and DWI for all participants were inspected by two authors (FX and KW) and 22 participants with excessive head motion and missing image data were excluded. The analysis sample of this study included 207 participants, which consisted of 113 patients including 38 adults with SZ (mean age = 35.68 ± 9.32 years; F/M = 9/29), 40 adults with BD (mean age = 34.50 ± 8.95 years; F/M = 18/22), and 35 adults with ADHD (mean age = 32.11 ± 10.45 years; F/M = 16/19), and 94 healthy participants (mean age = 31.56 ± 8.13 years; F/M = 43/51). Demographic and clinical information of the analysis sample is shown in Table 1.

**TABLE 1:** Demographic variables and clinical characteristics of all participants.

Variables	HC (*n* = 94)	SZ (*n* = 38)	BD (*n* = 40)	ADHD (*n* = 35)	χ^2^/*F*	*p*
Age (SD)	31.56 (8.13)	35.68 (9.32)	34.50 (8.95)	32.11 (10.45)	2.44	0.065
Gender (Male, %)	51 (54.25%)	29 (76.32%)	22 (55.00%)	19 (54.28%)	6.12	0.106
**Medication**						
Antipsychotics (*N*, %)	–	34 (89.5%)	19 (47.5%)	1 (2.9%)	54.79	<0.001
Antidepressants (*N*, %)	–	14 (36.8%)	14 (35%)	3 (8.6%)	9.10	<0.001
Mood stabilizer (*N*, %)	–	5 (13.2%)	26 (65%)	1 (2.9%)	41.99	<0.001
Psycho-stimulant (*N*, %)	–	0	5 (12.5%)	12 (34.3%)	17.07	<0.001
**BPRS score**						
Positive (*M*, SD)	–	2.94 (1.18)	1.29 (0.30)	1.11 (0.21)	74.35	<0.001
Negative (*M*, SD)	–	1.90 (0.76)	1.38 (0.52)	1.14 (0.28)	18.01	<0.001
Manic-hostility (*M*, SD)	–	1.64 (0.65)	1.94 (0.69)	1.75 (0.40)	2.47	0.089
Anxiety-depression (*M*, SD)	–	2.31 (1.13)	2.86 (1.22)	2.26 (0.83)	3.62	0.03
YMRS total score (SD)	–	7.79 (6.43)	11.2 (10.69)	5.94 (4.08)	4.52	0.013
HAMD total score (SD)	–	14.32 (11.81)	17.30 (12.71)	9.80 (6.08)	4.57	0.012
ASRS total score (SD)	8.73 (2.95)	9.58 (4.04)	13.05 (5.00)	15.43 (3.91)	32.78	<0.001

ADHD, attention-deficit/hyperactivity disorder; BD, bipolar disorder; SZ, schizophrenia; HC, Healthy Controls; ASRS, Adult ADHD Self-Report Scale; BPRS, Brief Psychiatric Rating Scale; HAMD, Hamilton Depression Rating Scale; YMRS, Young Mania Rating Scale.

All participants were aged 21–50 years and right-handed. As shown in the data descriptor (1), all of them were interviewed using the Diagnostic and Statistical Manual of Mental Disorders, Fourth Edition, and were based on the Structured Clinical Interview for DSM-IV supplemented by the Adult ADHD Interview (a structured interview form derived from the Kiddie Schedule for Affective Disorders and SZ, Present and Lifetime Version). ADHD criteria were assessed using Adult ADHD Interview.

Exclusion criteria of healthy group were any lifetime diagnosis of major psychiatric disorder. For the patient groups (SZ, BD, and ADHD), patients with other diagnoses or having used stable medications were excluded. For MRI scans, we excluded participants who were left-handed, who were pregnant, and those had other contraindications to scanning (e.g., claustrophobia, metal in the body). The UCLA CNP Study was approved by the UCLA institutional review board and was conducted following the Declaration of Helsinki. The current study was approved by the institutional review boards at the Shandong Normal University.

### Symptom assessment

Clinical symptoms in patient groups were assessed using clinical rating scales and self-report questionnaires. Psychotic symptoms were evaluated using the Brief Psychiatric Rating Scale (BPRS). Affective states were assessed with the Young Mania Rating Scale (YMRS) and Hamilton Psychiatric Rating Scale for Depression (HAMD). The adult ADHD Self-Report Scale (ASRS) was a self-report screening scale for ADHD. For more details about these four scales, see Supplementary Material.

### Magnetic resonance imaging acquisition parameters

MRI data were obtained using 3-T Siemens Trio scanner, which was located at UCLA, with the parameters for the T1-weighted high-resolution structural MPRAGE images: slice thickness, 1 mm; 176 slices; repetition time (TR), 1900 ms; echo time (TE), 2.26 ms; matrix, 256 × 256; FOV, 250 × 250 mm^2^. DWI were scanned with the following parameters: 64 directions; slice thickness, 2 mm; TR, 9000 ms; TE, 93 ms; 1 average; matrix, 96 × 96; flip angle, 90 degree; *b*-value, 1000 s/mm^2^.

### Image processing

All the DWI images were preprocessed by the VISTA pipeline,^2^ consisting of eddy-current correction, motion correction, T1 image alignment. Then, reconstruction of tractography was performed using the freely available AFQ package,^3^ which was a MATLAB toolbox and used to identify and quantify WM tracts in each subject’s brain. AFQ is a multi-step reconstructing procedure, which includes (1) all fibers are tracked within a WM mask using deterministic tractography (47); (2) each fiber was then qualified by using Wakana’s waypoint ROI procedure (48); (3) fiber tract refinement was performed according to the track probability maps (49); (4) after refinement, fiber tract cleaning applies the outlier rejection algorithm (47); (5) quantification of the diffusion measurements along each fiber track at 100 equidistant nodes. The FA values were averaged in cross-sections (i.e., segments) along the WM tracts (42) in each participant. While we focused on the SLF in this study, additional 18 major WM tracts were also examined, including bilateral anterior thalamic radiation (ATR), corticospinal tract (CST), cingulum cingulate (CCing), cingulum hippocampus (CHippo), inferior frontal-occipital fasciculus (IFOF), inferior longitudinal fasciculus (ILF), uncinated fasciculus (UF), arcuate fasciculus (AF), and corpus callosum (CC) (48). For a detailed characterization of FA along the SLF, we used the 100 segments as prior studies suggested (42, 45, 46).

### Statistical analysis

All statistical analyses were performed using IBM SPSS Statistics 26. Demographic and clinical variables were evaluated by analysis of variance and χ^2^ analysis between diagnostic groups. Using a three-way mixed-design analysis of covariance (ANCOVA), we examined the main effect of four groups on FA of the left and right SLF. Particularly, LATERALITY (1. Left; 2. Right) and SEGMENTS (1. 1–50 segments; 2. 51–100 segments) were regarded as within subject factors. Then, *post-hoc* comparisons were tested across these four groups. Finally, partial correlation analyses were conducted to determine the correlations between the mean FA values of each part of the SLF (1. 1–50 segments of left SLF; 2. 51–100 segments of left SLF; 3. 1–50 segments of right SLF; 4. 51–100 segments of right SLF) and seven clinical symptoms in patient groups. Given the effect of age and gender on WM, we included them as covariates in all above analyses. Here, we corrected the significance with Bonferroni method for multiple comparisons. The significance threshold was set at *p* = 0.05/7.

### Additional analyses

To validate our main findings, we firstly examined influence of medication status on the tracts. In each patient group, we classified subjects into subgroups according to their medication status (i.e., use of antipsychotics or other treatments for SZ; use of mood stabilizers or other treatments and antidepressants or other treatments for BD; and the use of psycho-stimulants of other treatments for ADHD). Then, two-sample *t*-tests were performed between each of the corresponding pairs of subgroups for the FA values of segments.

Second, in our current sample, 16 patients were diagnosed comorbidity with a secondary diagnosis (SZ patients comorbid with ADHD, *n* = 6; BD patients comorbid with ADHD, *n* = 7; SZ patients comorbid with BD, *n* = 2; ADHD patients comorbid with BD, *n* = 1; no patients comorbid with SZ). To partial out the impact of comorbidity, we excluded these 16 patients and performed the same statistical analyses to verify the results.

## Results

### Demographic and clinical characteristics

Demographic and clinical characteristics of the four groups are shown in Table 1. There were no significant differences in age or gender among the SZ, BD, ADHD, and HC groups. As expected, significant differences were observed in positive symptom, negative symptom and anxiety-depression among the three patient groups (all *p*_*s*_ < 0.05).

### Differences in fractional anisotropy between patient groups

After accounting for the effects of age and gender, a three-way mixed-design ANCOVA revealed a significant main effect of GROUP (between-subject effect: *F* [3, 201] = 3.687; *p* = 0.013) on FA in the second part (51–100 consecutive segments) of bilateral SLF. This analysis also revealed a significant effect of LATERALITY (within-subjects effect; *F* [1, 201] = 16.028; *p* < 0.05) and an effect of SEGMENT (within-subjects effect; *F* [49, 9849] = 36.192; *p* < 0.05). There were no significant group differences in the first part (1–50 consecutive segments) of SLF (Table 2).

**TABLE 2:** Three-way mixed design analysis of covariance (ANCOVA) in Superior longitudinal fasciculus in three kinds of psychiatric disorders and healthy controls.

	1–50 segments		51–100 segments	
Tests of between-subjects effects	*F* (3, 201)	*P*	*F* (3, 201)	*P*
Group	2.238	0.085	3.687	0.013
Tests of within-subjects effects	*F* (1, 201)	*P*	*F* (1, 201)	*P*
Laterality	1.776	0.184	16.028	<0.001
Laterality x group	1.349	0.260	0.830	0.479
Tests of within-subjects effects	*F* (49, 9849)	*P*	*F* (49, 9849)	*P*
Segment	31.762	<0.001	36.192	<0.001
Segment*group	1.314	0.018	1.128	0.175

Mauchly’s Test of Sphericity is used for the within-subject effect test in ANCOVA. Age and gender were covariates in all analyses.

**FIGURE 2:**
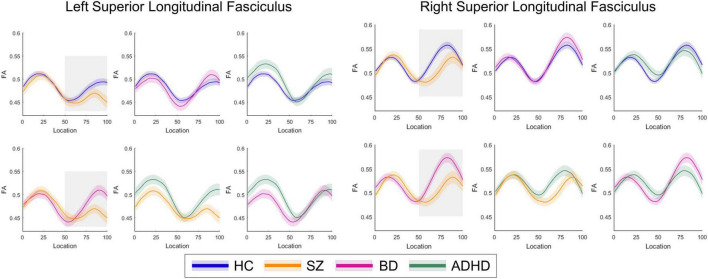
The plots of fractional anisotropy (FA) profiles of bilateral superior longitudinal fasciculus from healthy control individuals and patient subgroups.

**FIGURE 3:**
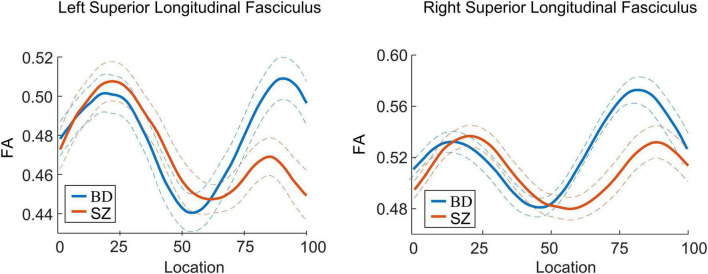
Curve-trajectory plots represent the mean fractional anisotropy (FA) along the left and right superior longitudinal fasciculus in schizophrenia (SZ) vs. bipolar disorder (BD).

*Post-hoc* analyses revealed that patients with SZ exhibited lower FA in the bilateral SLF than patients with BD in 51–100 segments (*p* < 0.05). There were no significant differences in FA between patients with BD and ADHD and healthy controls (*p*_*s*_ > 0.05) (Figures 2, 3). Additionally, analyses for FA values of another 18 WM tracts found no significant differences between groups.

### Clinical relationships

Partial correlation analyses were carried out to assess the relationships between mean FA values of the SLF and clinical characteristics in each patient group and all patients. Analyses across all patients found a significantly positive relationship between the mean FA value in the second part (51–100 segments) of the right SLF and the manic-hostility score of the BPRS (*r* = 0.285, *p* < 0.05). This positive association was also significant in patients with SZ (*r* = 0.391, *p* < 0.05). In patients with BD, higher mean FA values in the second part (51–100 segments) of the left SLF were associated with higher negative scores of the BPRS (*r* = 0.323, *p* < 0.05). No significant association was found in the ADHD group.

### Additional analyses

First, significant differences were observed in the first and second parts of the left SLF in patients with BD who had used mood stabilizers and other treatments. In SZ and ADHD groups, we did not observe any significant differences between unmedicated and medicated statuses (Supplementary Table 1).

Analyses excluding the 16 patients with comorbidity revealed same results. We found a significant main effect of GROUP (between-subject effect: *F* [3, 185] = 4.086; *p* = 0.008) on FA in the second part (51–100 consecutive segments) of bilateral SLF, and significant effects of LATERALITY (within-subjects effect; *F* [1, 185] = 14.050; *p* < 0.05) and SEGMENT (within-subjects effect; *F* [49,9065] = 33.449; *p* < 0.05). No significant group differences were found in the first part (1–50 consecutive segments) of SLF (Supplementary Table 2).

## Discussion

The present study aimed to examine whether the WM bundles were focal or extended in patients with SZ, BD, and ADHD by using the tract-profile approach. We found lower collinearity of fibers in posterior bilateral SLF tracts in adults with SZ compared to the healthy group, but no differences in adults with BD or ADHD. Comparisons between disorders revealed that patients with SZ had lower FA in half of parts of bilateral SLF compared to those with BD. Additionally, FA of the posterior right SLF was correlated with the manic-hostility score of the BPRS across all patients.

White matter impairments in patients with SZ were found in SLF, which was in line with prior studies (50–53). Our findings further demonstrated that lower FA in the SLF was focal, not in the entire tract. Reduced FA may indicate impaired myelination, altered fiber organization, or aberrant axon morphology. The focal abnormalities in this tract probably reflected abnormalities in the collinearity/integrity of specific fibers that connected the frontal lobe with occipital, parietal, and temporal lobes. Regarding BD patients, a prior study found a trend toward increased FA in posterior of bilateral SLF compared to healthy controls (27). Here, we showed that patients with SZ had lower FA in the left and right posterior SLF when compared to BD patients. By contrast, a previous study reported no significant group differences in structural connectivity of SLF between SZ and BD groups (27). The difference in patient characteristics such as age, gender, medical status, and the tractography method in the previous study may be reasons for the inconsistency. Further research using various tractography methods and a homogenous patient sample are required to verify findings of this study. Given the results of our study, SLF may be a distinct neural marker for the two disorders, and this tract might be more affected and disrupted in SZ than in BD. A previous diffusion study has employed similar tractography approaches and compared children patients with BD and patients with ADHD (11). As in this study, the authors found no diffusion imaging abnormalities in the SLF. In fact, within the attentional network model, the microstructure of the SLF might be an important mechanism underlying poor attention in patients with ADHD (54). Further research with a larger sample size is needed to elucidate the distinction in SLF between ADHD and healthy group.

We also found that higher FA values in the posterior right SLF were associated with higher manic-hostility scores across the patients. This suggested that the partial SLF abnormalities may contribute to manic-hostility symptoms. A previous study reported that greater thickness in the frontoparietal network was positively correlated with hostility score in patients with BD (55). There was no published literature on the association between manic-hostility symptoms and microstructural changes of SLF in mental disorders. Our research might serve as an explorative finding, and potential associations warrant further research.

Several limitations should be considered. First, this is a cross-sectional study and thus the causal relationship between focal SLF abnormalities and SZ remains to be explored. Longitudinal research are wanted in future to examine the development trajectories of SLF tracts among these psychiatric disorders. Second, impact of the age of onset has not been considered due to lack of data. Kelly et al. (15) found that the duration of illness could be an important factor in WM impairments in SZ. They also found that several brain regions showed a negative association between FA values and duration of illness. Third, type of medications might influence the microstructural integrity of the WM tracts. A prior study found that a higher proportion of patients taking antipsychotics was associated with smaller FA reduction in patients with first-episode psychosis (56). Although we examined impact of medication and did not find any significant differences in SZ, our results need to be validated in future studies in drug-naive first-episode patients.

In summary, using a tract-profile approach in patients with SZ, BD, and ADHD, we demonstrated focal abnormalities in the posterior of the SLF in patients with SZ. In contrast, no significant differences were found in patients with BD and ADHD when compared to the healthy group. The posterior of the SLF links the regions between frontal and parietal cortices and has been proposed to have a key role in the pathophysiology of SZ. Correlation between focal abnormalities of the right SLF and the manic-hostility symptom was also found. Our finding may encourage further research on focal WM abnormalities and the common and distinct pathophysiology of psychiatric disorders. Future research is needed to validate our results and further examine the effect of confounding factors such as medications and duration of illness.

## Data availability statement

The datasets presented in this study can be found in online repositories. The names of the repository/repositories and accession number(s) can be found below: http://openfmri.org.

## Ethics statement

The studies involving human participants were reviewed and approved by the Institutional Review Board of the Shandong Mental Health Center. The patients/participants provided their written informed consent to participate in this study. Written informed consent was obtained from the individual(s) for the publication of any potentially identifiable images or data included in this article.

## Author contributions

KW and YY designed the study. KW, FX, and CJ analyzed the data. FX and CJ drafted the manuscript. KW, YY, FX, CJ, TZ, and RW revised the manuscript. All authors approved the final version of the manuscript.

## Footnotes

1
http://openfmri.org

2
https://web.stanford.edu/group/vista/cgi-bin/wiki/index.php/DTI_Preprocessing

3
https://github.com/yeatmanlab/AFQ/wiki
