# Supplementary material for: Segmental abnormalities of superior longitudinal fasciculus microstructure in patients with schizophrenia, bipolar disorder, and attention-deficit/hyperactivity disorder: An automated fiber quantification tractography study
Source: Front Psychiatry. 2022 Dec 6;13:999384. doi: 10.3389/fpsyt.2022.999384 (PMC9766353; doi:10.3389/fpsyt.2022.999384)
Supplement: Supplementary file 1 [file Data_Sheet_1.docx]

**Segmental abnormalities of superior longitudinal fasciculus microstructure in patients with schizophrenia, bipolar disorder, and ADHD: an automated fiber quantification tractography study**

Feiyu Xu, Chengliang Jin, Tiantian Zuo, Ruzhan Wang, Ying Yang, Kangcheng Wang

**Supplemental Method**

**1.1 The Brief Psychiatric Rating Scale (BPRS)**

The Brief Psychiatric Rating Scale (BPRS) is a 24-item clinician-rated measure that assesses clinical psychiatric symptoms (e.g., somatic concern, suicidality, unusual thought content) experienced over the previous week (1, 2). Items are rated on a 7-point Likert scale, ranging from 1 (not present) to 7 (extremely severe). Four subscale scores (Positive Symptoms, Negative Symptoms, Manic-Hostility, Anxiety- Depression) are used to assess the current psychopathology and psychotic symptoms. The scale is widely used, and its reliability and validity have been found to be good in adult samples (3).

**1.2 Young Mania Rating Scale-C (YMRS)**

Young Mania Rating Scale-C (YMRS) is an 11-item clinical-rated measure that assesses the main symptoms of the manic episode in terms of mood, mobility, sexual desire, sleep, irritability, speech, flight of ideas, grandiosity, aggressive behaviors, appearance, and one item that assesses the patient’s insight (4). Seven items are rated on a severity scale, ranging from 0 to 4, and the other four items (irritability, speech, thought content, and aggressive-destructive behaviors) scores range from 0 to 8, with higher numbers indicating greater severity of the symptom. The total score of the scale is calculated by summing all items. Good reliability and validity of the YMRS were found in adult populations (5).

**1.3 Hamilton Psychiatric Rating Scale for Depression (HAMD)**

Hamilton Psychiatric Rating Scale for Depression (HAMD) (6) is a clinician-administered rating scale to assess symptom severity. Each item is rated on either a 0-4 or 0-2 scale. The items are ranging from 0 to 4, with the higher numbers indicating greater severity of the symptom. The other items are ranging from 0 to 2, with the quantification of severity being difficult, and thus the item is rated as either probably or definitely present. The total score of the scale is calculated by summing all items. The higher the score, the more severe the manifestation of a depressive symptom. The reliability of HAMD was highly acceptable as in previous studies (7).

**1.4 The Adult ADHD Self-Report Scale (ASRS)**

The Adult ADHD Self-Report Scale (ASRS v1.1) is an 18-item self-report questionnaire designed to assess ADHD symptoms in adults (8). Six of the eighteen questions are found to be the most predictive of symptoms consistent with ADHD. These six questions are the basis for the ASRS v1.1 Screener and are also Part A of the Symptom Checklist. Part B of the Symptom Checklist contains the remaining twelve questions. All 18 items are ranging from 0 (never) to 4 (very often). For the sum score of the scale, we summed all 18 items. The ASRS have demonstrated good psychometric properties in studies with adults (9, 10).

**Supplemental Tables**

Table S1. Effects of taking distinct medication or not on FA of segments SLF in each patient group.

| Segments of SLF | SZ | BD | | ADHD |
| --- | --- | --- | --- | --- |
|  | Comparison of antipsychotics with other treatments | Comparison of antidepressants with other treatments | Comparison of mood stabilizers with other treatments | Comparison of psycho-stimulant with other treatments |
| 1-50 segments of left | 0.035 (0.304) | 0.845 (0.921) | 1.564 (0.015) | 0.004 (0.627) |
| 51-100 segments of left | 0.024 (0.552) | 0.16 (0.408) | 1.132 (0.02) | 0.003 (0.21) |
| 1-50 segments of right | 0.173 (0.445) | 0.136 (0.675) | 0.011 (0.77) | 0.917 (0.755) |
| 51-100 segments of right | 0.174 (0.237) | 0.648 (0.241) | 0.748 (0.939) | 0.472 (0.257) |

Data are presented as T-values (P-values). Only in the first half (1-50 segments), there was significant differences between patients with mood stabilizers treatment and other treatments. No significant result was not obtained in the groups of SZ and ADHD. SLF, superior longitudinal fasciculus; SZ, schizophrenia; BD, bipolar disorder; ADHD, attention-deficit/hyperactivity disorder.

Table S2. Three-way mixed design ANCOVA in Superior longitudinal fasciculus in three kinds of psychiatric disorders and healthy control, after excluding the 16 patients comorbid with any disorders of ADHD, BD and SZ.

|  | 1-50 segments | | 51-100segments | |
| --- | --- | --- | --- | --- |
| Tests of between-subjects effects | F (3,185) | p | F (3,185) | p |
| Group | 1.876 | 0.135 | 4.086 | 0.008 |
| Tests of within-subjects effects | F (1,185) | p | F (1,185) | p |
| Laterality | 2.053 | 0.184 | 14.050 | <0.001 |
| Laterality*group | 1.664 | 0.176 | 0.409 | 0.747 |
| Tests of within-subjects effects | F (49,9065) | p | F (49,9065) | p |
| Segment | 30.844 | <0.001 | 33.449 | <0.001 |
| Segment*group | 1.318 | 0.018 | 1.084 | 0.269 |

Mauchly's Test of Sphericity is used for the within-subject effect test in ANCOVA. Age and gender were covariates in all analyses. ADHD, attention-deficit/hyperactivity disorder; BD, bipolar disorder; SZ, schizophrenia.

**References**

1. Ventura J, Green MF, Shaner A, Liberman RP. Training and quality assurance with the Brief Psychiatric Rating Scale: "The drift busters.". International Journal of Methods in Psychiatric Research. 1993;3(4):221-44.

2. Overall JE, Gorham DR. The Brief Psychiatric Rating Scale. Psychological Reports. 1962;10(3):799-812.

3. Thomas A, Donnell AJ, Young TR. Factor Structure and Differential Validity of the Expanded Brief Psychiatric Rating Scale. Assessment. 2004;11(2):177-87.

4. Double DB. The factor structure of manic rating scales. J Affect Disord. 1990;18(2):113-9.

5. Young RC, Biggs JT, Ziegler VE, Meyer DA. A rating scale for mania: reliability, validity and sensitivity. Br J Psychiatry. 1978;133:429-35.

6. Hamilton M. A rating scale for depression. J Neurol Neurosurg Psychiatry. 1960;23(1):56-62.

7. Trajković G, Starčević V, Latas M, Leštarević M, Ille T, Bukumirić Z, et al. Reliability of the Hamilton Rating Scale for Depression: a meta-analysis over a period of 49 years. Psychiatry Res. 2011;189(1):1-9.

8. Kessler RC, Adler L, Ames M, Demler O, Faraone S, Hiripi E, et al. The World Health Organization Adult ADHD Self-Report Scale (ASRS): a short screening scale for use in the general population. Psychol Med. 2005;35(2):245-56.

9. Adler LA, Spencer T, Faraone SV, Kessler RC, Howes MJ, Biederman J, et al. Validity of pilot Adult ADHD Self- Report Scale (ASRS) to Rate Adult ADHD symptoms. Ann Clin Psychiatry. 2006;18(3):145-8.

10. Kessler RC, Adler LA, Gruber MJ, Sarawate CA, Spencer T, Van Brunt DL. Validity of the World Health Organization Adult ADHD Self-Report Scale (ASRS) Screener in a representative sample of health plan members. Int J Methods Psychiatr Res. 2007;16(2):52-65.
